# CpG Island Methylator Phenotype, *Helicobacter pylori*, Epstein-Barr Virus, and Microsatellite Instability and Prognosis in Gastric Cancer: A Systematic Review and Meta-Analysis

**DOI:** 10.1371/journal.pone.0086097

**Published:** 2014-01-27

**Authors:** Liang Zong, Yasuyuki Seto

**Affiliations:** Department of Gastrointestinal Surgery, Graduate School of Medicine, University of Tokyo, Tokyo, Japan; Institut Pasteur Paris, France

## Abstract

**Background:**

The controversy of CpG island methylator phenotype (CIMP) in gastric cancer persists, despite the fact that many studies have been conducted on its relation with *helicobacter pylori* (H. *pylori*), Epstein-Barr virus (EBV), and microsatellite instability (MSI) and prognosis. To drive a more precise estimate of this postulated relationship, a meta-analysis was performed based on existing relevant studies.

**Methods:**

We combined individual patient data from 12 studies which involved 1000 patients with gastric cancer, which met the criteria. We tabulated and analyzed parameters from each study, including H. *pylori*, EBV, MSI, and clinical information of patients.

**Results:**

The overall OR for H. *pylori* infection in CIMP positive group *vs.* negative group revealed that significantly elevated risks of positive H. *pylori* infection in the former were achieved (OR 2.23 95% CI, 1.25–4.00; P = 0.007, P_heterogeneity_ = 0.05). Similarly, strong relation between EBV infection and CIMP was achieved by OR 51.27 (95% CI, 9.39–279.86; P<0.00001, P_heterogeneity_ = 0.39). The overall OR for MSI in CIMP positive group *vs.* negative group was 4.44 (95% CI, 1.17–16.88; P = 0.03, P_heterogeneity_ = 0.01). However, there did not appear to be any correlations with clinical parameters such as tumor site, pathological type, cell differentiation, TNM stage, distant metastasis, lymph node metastasis, and 5-year survival.

**Conclusions:**

The meta-analysis highlights the strong relation of CIMP with H. *pylori*, EBV, and MSI, but CIMP can not be used as a prognostic marker for gastric cancer.

## Introduction

Gastric cancer is the fourth most common malignancy and second leading cause of cancer death in the world [1]. Even screening programs with barium photofluorography or endoscopy allow earlier detection in Japan and Korea, the patients with advanced gastric cancer are still worse in 5-year overall survival. Identifying molecular aberrations in gastric cancer may improve our understanding of gastric carcinogenesis, identify strategies for subdividing patients into relevant subgroups, and highlight novel molecular target agents. Although the molecular mechanisms of gastric cancer carcinogenesis remain unclear, both genetic and epigenetic alterations are important. Genetic alterations are responsible for activation of onceogenes and inactivation of tumor-suppressor gene. Epigenetic alteration through DNA methylation is known to play an important role in inhibiting the expression of tumor-related genes.

Aberrant DNA methylation in cancer was summarized as global hypomethylation and regional hypermethylation, which are associated with genomic instability and inactivation of tumor-suppressor genes, respectively [2]. However, regional hypermethylation refers to the aberrant methylation of normally unmethylated sequences, most of which are clusters of CpG sites, denoted CpG island. Because multiple genes are concurrently methylated in the hypermethylated subtype, CIMP concept was first introduced to the molecular pathways of colorectal cancers by Dr. Issa group [3]. After that, Scientists have found that CIMP-positive colorectal cancers have a close association with the molecular and clinicopathological features, and poor prognosis [4]–[6].

Similarly, the presence of CIMP-positive gastric cancer has been reported by many scientists [7]–[24], but controversial data did not confirm the prognostic value of CIMP for gastric cancer. This was possibly due to small sample size or confounding variables. Therefore, we initiated an international collaborative effort which resulted in a meta-analysis of data on individual patient in prospective cohort studies to evaluate the association between CIMP and malignant behavior in gastric cancer.

## Methods

### Publication Search

Two electronic databases (PubMed and Embase) were searched (last search was updated on 21 November 2012, using the search terms: ‘Gastric cancer’ and ‘CIMP’. All eligible studies were retrieved, and their bibliographies were checked for other relevant publications. Review articles and bibliographies of other relevant studies identified were hand-searched to identify additional eligible studies. Only published studies with full-text articles were included. When the same patient population was included in several publications, only the most recent or complete study was used in this meta-analysis.

### Inclusion Criteria

The inclusion criteria were as follows: (a) evaluating the relation between CIMP and H. *pylori*, EBV, MSI or clinical prognostic parameters; (b) promoter methylation; and (c) sufficient published data to estimate an odds ratio (OR) with 95% confidence interval (CI).

### Data Extraction

Information was carefully extracted from all eligible studies by two of the authors (Zong L and Seto Y), according to the inclusion criteria listed above. The following data were collected from each study: first author's surname, publication date, study method, sample size, total number of patients with positive CIMP and negative CIMP, and number of patients divided by age, gender, tumor site, pathological type, cell differentiation, TNM stage, lymph node metastasis, distant metastasis and 5-year overall survival in those with and without CIMP, respectively. We did not define a minimum number of patients for inclusion in our meta-analysis.

### Statistical Analysis

Odd ratios with 95% CI were used to assess the correlation of CIMP with H. *pylori*, EBV, and MSI and prognosis, according to the method of Woolf. Heterogeneity assumption was confirmed by the X^2^-based Q-test. A P-value greater than 0.10 for the Q-test indicated a lack of heterogeneity among the studies, therefore, the OR estimate for each study was calculated by the fixed-effects model. Otherwise, the random-effects model was used. The significance of the pooled OR was determined by the Z-test and P>0.05 was considered statistically significant. Sensitivity analyses were carried out to determine if modification of the inclusion criteria for this meta-analysis affected the final results. An estimate of potential publication bias was carried out using the funnel plot, in which the OR for each study was plotted against its log (OR). An asymmetric plot suggested possible publication bias. Funnel plot asymmetry was assessed using Egger's linear regression test, a linear regression approach to measure funnel plot asymmetry on the natural logarithm scale of the OR. The significance of the intercept was determined by the t-test, as suggested by Egger (P<0.05 was considered representative of statistically significant publication bias). All statistical tests were performed with Review Manager Version 5.0 (The Cochrane Collaboration, Oxford, England).

## Results

### Study Characteristics

**Figure 1:**
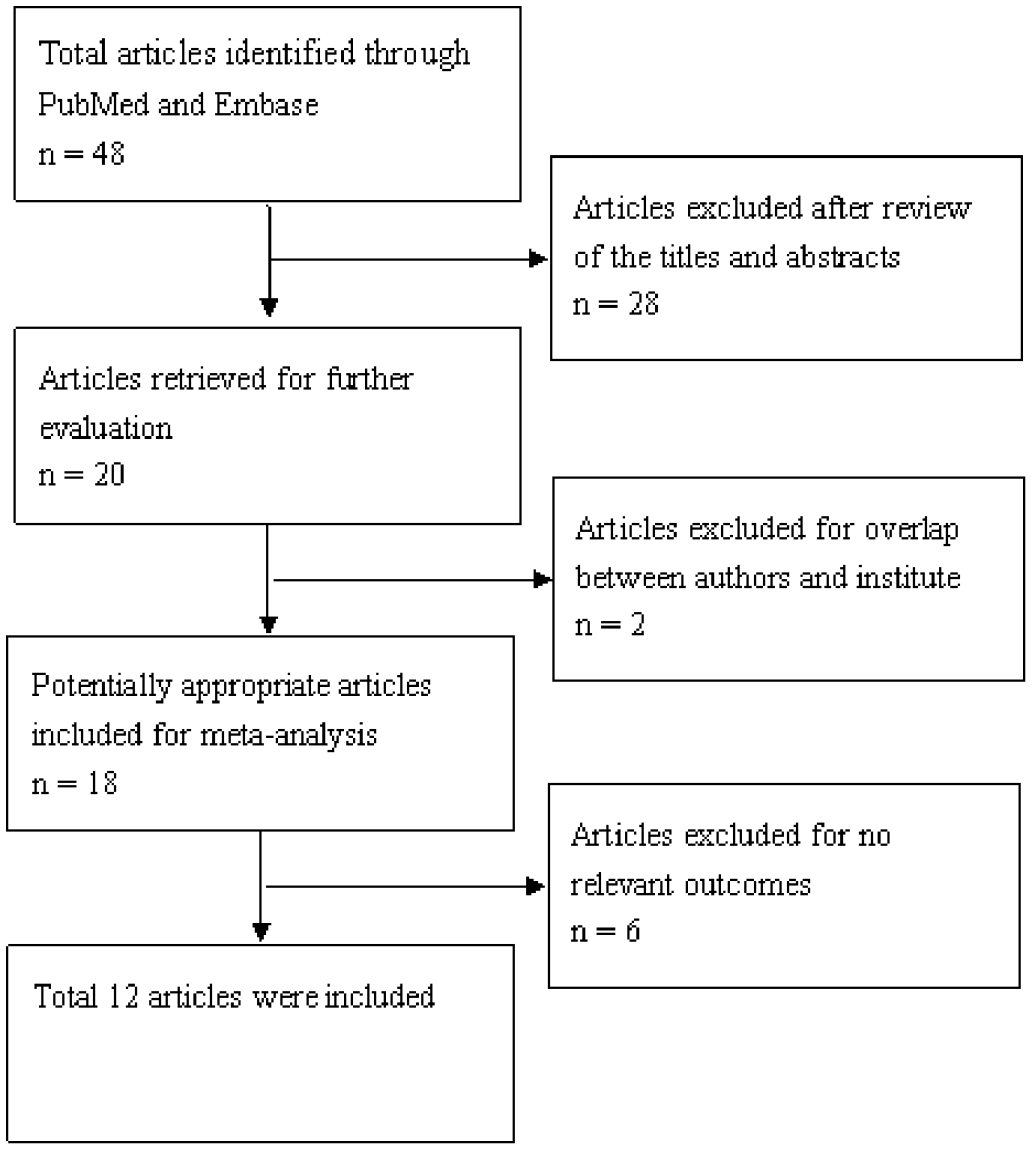
Flow chart of literature selection.

A total of 18 publications met the basic inclusion criteria [7]–[24]. The study by Oue *et al* was excluded because they did not categorized into CIMP subgroups with methylated gene panel [7]. In addition, the study by Kanai *et al* was excluded because they focused on DNA methylation of CPG islands and pericentromeric satellite regions in colorectal and stomach cancer [8]. Similarly the study by Oshimo *et al* was excluded because they mainly analyzed the relation between epigenetic inactivation of RIZ1 and CIMP [9]. The study by Watanabe *et al* was not included because they tried to prove fidelity in replicating DNA methylation patterns in cancer cells lead to dense methylation of a CpG island [10]. Other studies were excluded due to insufficient information to calculate OR [11], [12]. Hence, a total of 12 studies including 1000 patients were used in the pooled analyses. Table 1 lists the studies identified and their main characteristics. Of the 12 groups, sample size ranged from 40 to 200 (Figure 1).

**Table 1:** Main characteristics of all studies included in the meta-analysis.

Study	Study Period	N	Study Method	CpG island site	Gene type	Cut off value	Features
Kim *et al*	2003	200	MSP	Promoter	MINT1, MINT2, MINT12, MINT25, MINT31	CIMP-high (3–5), CIMP-intermediate (2), CIMP-low (1 or less)	MSI-H GC was related to the high CIMP.
An *et al*	2005	82	MSP microdissection	Promoter	MINT1, MINT2, MINT25, MINT31, MLH1	CIMP-high (3 or more), CIMP-low (2 or less), CIMP-negative (0)	CIMP-high GC was associated with MSI and better prognosis.
Kim *et al*	2005	40	MSP	Promoter	hMLH1, MINT2, TIMP3, THBS1, DAP-K, GST P1, APC	CIMP-high (3–7), CIMP-low(1–2), CIMP-negative (0)	There were no specific findings for CIMP-high GC.
Oue *et al*	2006	75	COBRA	Promoter	MINT1, MINT2, MINT12, MINT25, MINT31	CIMP+ (3 or more), CIMP− (2 or less)	There were no specific findings for CIMP+ GC.
Chang *et al*	2006	91	MSP	Promoter	LOX, HRASLS, FLNc, HAND1, TM	CIMP-high (4–5), CIMP-intermediate (1–3), CIMP-negative (0)	CIMP-high GC was associated with better prognosis.
Kusano *et al*	2006	78	COBRA	Promoter	MINT1, MINT2, MINT12, MINT25, MINT31	CIMP-high (4–5), CIMP-intermediate (1–3), CIMP-negative (0)	CIMP-high GC was closely associated with proximal location, diffuse type, less-advanced tumor stage, and better prognosis.
Enomoto *et al*	2007	66	MSP	Promoter	LOX, HRASLS, FLNc, HAND1,THBD, F2R, NT5E, GREM1, ZNF177, CLDN3, PAX6, CTSL	CIMP-high (5 or more), CIMP-low (1–4), CIMP-negative (0)	CIMP-high GC was closely associated with diffuse type and better prognosis.
Zhang *et al*	2008	47	MSP	Promoter	hMLH1, MINT1, MINT2, MINT31, p16	CIMP-high (3–5), CIMP-intermediate (1–2), CIMP-negative (0)	There were no specific findings for CIMP-high GC.
Kondo *et al*	2009	41	MSP	Promoter	hMLH-1, MINT1, MINT2, MINT31, Kip2, p16, p15, p73, MGMT, DAPK, HCAD	CIMP+ (4 or more), CIMP− (3 or less)	H. *pylori* infection caused the aberrant DNA hypermethylation of specific genes and induced CIMP.
Park *et al*	2010	150	MethyLight PCR	Promoter	BCL2, BDNF, CACNA1G, CALCA, CHFR, CYP1B1, DLEC1, GRIN2B, RUNX3, SEZ6L, SFRP4, TERT, THBS1, TIMP3, TP73, TWIST1.	CIMP-high (14–16), CIMP-low (1–3), CIMP-negative (0)	CIMP-high GC was featured with poor prognosis.
Chen *et al*	2012	120	MethyLight PCR	Promoter	ALX4, TMEFF2, CHCHD10, IGFBP3, NPR1	CIMP-high (4–5), CIMP-low (1–3), CIMP-negative (0)	CIMP-high GC was associated with more distant lymph node metastasis.
Liu *et al*	2012	72	MSP	Promoter	APC, WIF1, RUNX3, DLC1, SFRP1, DKK, E-cad	CIMP+ (3 or more), CIMP− (2 or less)	H. *pylori*+/CIMP+ cases were associated with higher rates of metastasis and recurrence than H. *pylori*+/CIMP− cases.

COBRA, combined bisulfite restriction analysis; CIMP, CpG island methylator phenotype; GC, gastric cancer; MSP, methylation-specific PCR; Q-MSP, quantitative MSP.

### Correlation with Virus Infection and Molecular Stability

**Figure 2:**
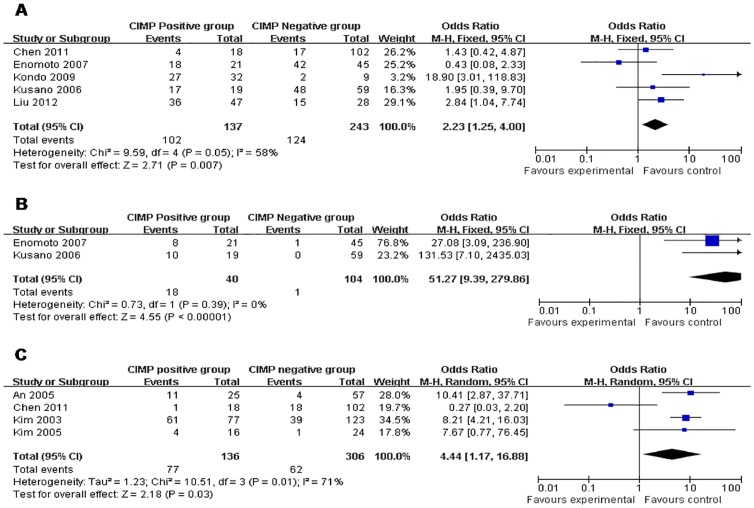
CIMP+ *vs.* CIMP−: a) H. *pylori*; b) EBV; c) MSI.

The overall OR for H. *pylori* infection in CIMP positive group *vs.* negative group revealed that significantly elevated risks of positive H. *pylori* infection in the former were achieved (OR 2.23 95% CI, 1.25–4.00;P = 0.007, P_heterogeneity_ = 0.05). Similarly, strong relation between EBV infection and CIMP was achieved by OR 51.27 (95% CI, 9.39–279.86; P<0.00001, P_heterogeneity_ = 0.39). The overall OR for MSI in CIMP positive group *vs.* negative group was 4.44 (95% CI, 1.17–16.88; P = 0.03, P_heterogeneity_ = 0.01) (Table 2 and Figure 2).

**Table 2:** Outcomes of the meta-analysis.

Parameters	No. Studies	Sample Size		Heterogeneity	OR	95% CI of Overall Effect	P
		CIMP+	CIMP−				
H. *pylori*	5	137	243	P = 0.05, I^2 = ^58%	2.23	1.25–4.00	P = 0.007
EBV	2	40	104	P = 0.39, I^2 = ^0%	51.27	9.39–279.86	P<0.00001
MSI	4	136	306	P = 0.01, I^2 = ^71%	4.44	1.17–16.88	P = 0.03
Age	4	87	195	P = 0.52, I^2 = ^0%	1.10	0.63–1.93	P = 0.74
Gender	9	184	565	P = 0.91, I^2 = ^0%	0.69	0.48–1.00	P = 0.05
Tumor site	4	85	206	P = 0.17, I^2 = ^40%	0.85	0.51–1.44	P = 0.55
Pathological type	7	142	440	P = 0.03, I^2 = ^56%	0.63	0.31–1.28	P = 0.20
Cell differentiation	3	45	264	P = 0.68, I^2 = ^0%	0.64	0.29–1.42	P = 0.27
TNM stage	4	47	212	P = 0.08, I^2 = ^56%	1.39	0.54–3.57	P = 0.49
Distant metastasis	4	103	258	P = 0.004, I^2 = ^77%	1.69	0.37–7.67	P = 0.49
Lymph node metastasis	6	128	349	P = 0.16, I^2 = ^37%	0.81	0.50–1.31	P = 0.39
5-year survival	2	32	114	P = 0.02, I^2 = ^82%	0.65	0.04–10.70	P = 0.76

CIMP, CpG island methylator phenotype; CI, confidence interval; EBV, Epstein-Barr virus; H. *pylori*, *Helicobacter pylori*; MSI, microsatellite instability; OR, odds ratio.

### Correlation with Clinical Information

**Figure 3:**
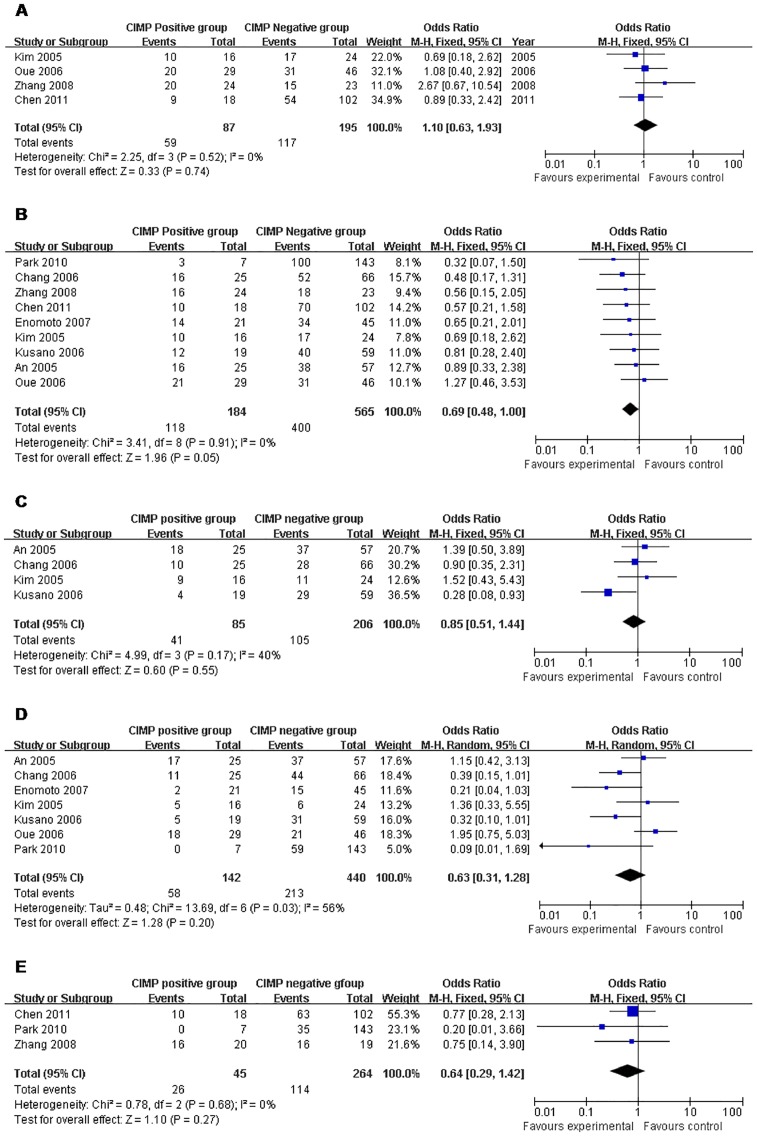
CIMP+ *vs.* CIMP−: a) Age; b) Gender; c) Tumor site; d) Pathological type; e) Cell differentiation.

The meta-analysis of both age distribution and gender in the CIMP-positive *vs.* -negative groups did not attain statistical significance (OR 1.10 95% CI, 0.63–1.93; P = 0.74, P_heterogeneity_ = 0.52) and (OR 0.69 95% CI, 0.48–1.00; P = 0.05, P_heterogeneity_ = 0.91). The overall OR for tumor site in the CIMP-positive *vs.* -negative subgroups was 0.85 (95% CI, 0.51–1.44; P = 0.55, P_heterogeneity_ = 0.17). The overall OR for either pathological type or cell differentiation in the CIMP-positive *vs.* -negative subgroups did not show any positive finding (OR 0.63 95% CI, 0.31–1.28; P = 0.20, P_heterogeneity_ = 0.03 and OR 0.64 95% CI, 0.29–1.42; P = 0.027, P_heterogeneity_ = 0.68, respectively) (Table 2 and Figure 3).

### Correlation with Prognostic Parameters

**Figure 4:**
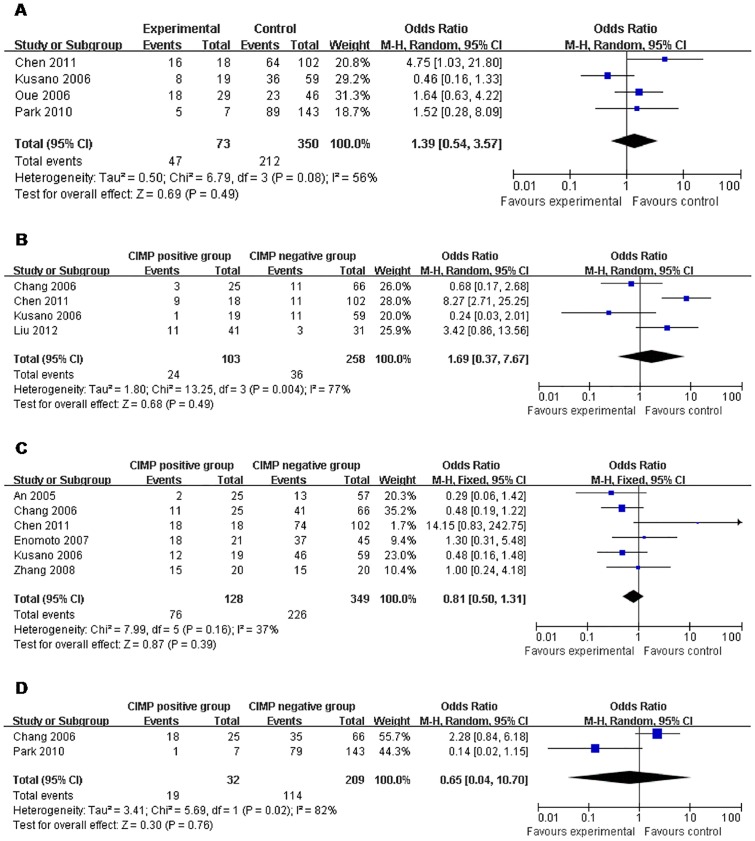
CIMP+ *vs.* CIMP−: a) TNM stage; b) Distant metastasis; c) Lymph node metastasis; d) 5-year survival.

Although some studies reported that CIMP may correlate with a better prognosis in gastric cancer, no matter on TNM stage, distant metastasis, lymph node metastasis, or even 5-year survival rate, CIMP did not have any significant correlation with one of them in our analysis (Table 2 and Figure 4).

### Publication Bias

Begg's funnel plot was performed to assess publication bias. The heterogeneity tests for comparing the 12 combined studies showed heterogeneity in some analyses such as MSI, pathological type, distant metastasis and 5-year survival. However, no single study influenced the pooled OR qualitatively as indicated by the sensitivity analyses (data not shown).

## Discussion

Epigenetic alterations have been suggested to be significant initiating events in cancerization [25]. However, with the deep involvement of aberrant DNA methylation in human cancers becoming clear, the occasional presence of aberrant DNA methylation in non-cancerous tissues was recognized in the Barrett's esophagus [26], stomach [27], colon [28], [29], and liver [30], which suggested the involvement of the former in the field for cancerization. Chronic inflammation, possibly specific types is likely to induce aberrant DNA methylation in normal tissues and thus form an “epigenetic field for cancerization”. To supply the evidence, it is well known that chronic inflammation plays an important role in ulcerative colitis for colon cancers, chronic hepatitis for liver cancers, and Barrett's esophagus for esophagus cancers. Infection by H. *pylori* is known to induce severe chronic inflammation, which were involved in the induction of the field for gastric cancers. In addition, aberrant DNA methylation in gastric biopsies from H. *pylori* ^+^ patients was found to be correlated with a greater gastric cancer risk in several studies [31], [32]. Therefore, aberrant DNA methylation might be the key event in tumor genesis of gastric cancer.

In last decade, careful quantitative evaluation showed that many genes that are highly methylated in carcinoma also show a low but measurable degree of methylation in normal mucosa [33]. Furthermore, aberrant methylated region focusing in the promoter rich in CpG islands suggested the key step in epigenetic gene silencing. It is therefore necessary to elucidate the methylation statuses of a panel of representative genes in an individual disease. To achieve this goal, CpG island methylator phenotype was introduced by Toyota *et al* [3]. Till now, it was well evidenced that CIMP is associated with poor prognosis in colorectal cancer, lung cancer and neuroblastoma. Although the term “CIMP” has been used in a variety of ways in the context of gastric cancer [18], its prognostic value in gastric cancer is still controversial. For example, An *et al.* showed that CIMP, which correlated with malignant features on histopathology, was an independent prognostic factor for overall and cause-specific survival in patients with gastric cancer [14], whereas Kim *et al.* and Zhang *et al.* failed to observe such an association [15], [20]. Park *et al.* suggested that CIMP-high GCs were featured with characteristic clinicopathological parameters, including poor prognosis [22]. However, in contrast, Chang *et al.* and Kusano *et al.* suggested that CIMP-high showing better prognosis [17], [18]. Some scientists supposed this discrepancy might come from using different CIMP marker panels as the determination of CIMP status; however, there was a common point of these studies in which the methylated cite of CIMP marker genes lied in promoter. Therefore, it still represents a trend of methylation level in promoter, by which the CIMP is meaningful in tumorigenesis of gastric cancer. In our opinion, prospective data was less convincing mainly due to small sample size and the lack of statistical power to integrate sporadic individual studies.

With the goal to explore the potential value of CIMP in gastric cancer, we performed this meta-analysis of published studies to derive an overall pooled estimation. Since some studies have divided patients into three groups, CIMP-high, CIMP-immediate, CIMP-low, we combine the latter two into CIMP-negative subtype in comparison with no changed CIMP-high as CIMP-positive subtype. From table 2, our findings strongly suggested that H. *pylori* and EBV infections cause the aberrant DNA hypermethylation of specific genes and induce CIMP, an important epigenetic mechanism of the tumorigenesis. However, the mechanism for aberrant DNA hypermethylation induced by H. *pylori* might be different from that by EBV. Recent study by Huang *et al.* supported that H. *pylori* infection causes gastric mucosal inflammatory responses, resulting in up-regulation of interleukin-1b (IL-1b) and overproduction of mutagenic nitric oxide (NO), by which aberrant DNA methylation was induced [34]. As for EBV infection, it was suggested that the methylation mechanism in host cells might be primarily for defense against foreign DNA and that the host-driven extensive methylation of viral genome may also trigger host genome methylation [35]. It was also demonstrated repeatedly that direct interaction of viral latent proteins with DNA methyl transferases (DNMT), up-regulation of DNMT genes by viral latent proteins, and increased expression of polycomb group proteins may contribute to alternations in DNA methylation and histone modifications [35]–[38].

Furthermore, strong relation of CIMP with MSI reveals that CIMP may have a potential relation with gene mutations, which may cooperate with each other in development and progression of gastric cancer. However, the meta-analysis did not show any correlations with clinical parameters such as age, gender, tumor site, pathological type, cell differentiation, TNM stage, distant metastasis, lymph node metastasis, and 5-year survival.

If CIMP was a key incidence in gastric cancer, the reason why the CIMP in promoter could not be used as a prognostic marker is not clear. It is possible that gene methylation in promoter lead to the primary tumor genesis but not progressing in gastric cancer. However, another possible reason is that limited methylated CpG island sites do not represent the true trend of CIMP. Regardless of the above analysis, heterogeneity is also one of the important sources that limited us to make more precise conclusion. Therefore, it is essential to develop more extensive large-scale study with bead-array technology in future.

## Supporting Information

Checklist S1 (DOC)

Diagram S1 (DOC)
