# Supplementary material for: CpG Island Methylator Phenotype, Helicobacter pylori, Epstein-Barr Virus, and Microsatellite Instability and Prognosis in Gastric Cancer: A Systematic Review and Meta-Analysis
Source: PLoS One. 2014 Jan 27;9(1):e86097. doi: 10.1371/journal.pone.0086097 (PMC3903497; doi:10.1371/journal.pone.0086097)
Supplement: Diagram S1 — (DOC) [file pone.0086097.s002.doc]

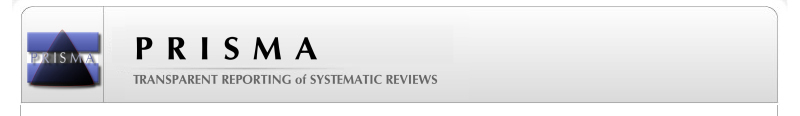
**PRISMA 2009 Flow Diagram**

**Screening**

**Included**

**Eligibility**

**Identification**

Records identified through database searching
(n = 28)

Additional records identified through other sources
(n =20 )

Records after duplicates removed
(n = 46 )

Records screened
(n = 20)

Records excluded
(n = 2 )

Full-text articles assessed for eligibility
(n = 18 )

Full-text articles excluded, with reasons
(n =6 )

Studies included in qualitative synthesis
(n = 12 )

Studies included in quantitative synthesis (meta-analysis)
(n = 12 )
